# An Effective Sensor Architecture for Full-Attitude Determination in the HERMES Nano-Satellites

**DOI:** 10.3390/s23052393

**Published:** 2023-02-21

**Authors:** Andrea Colagrossi, Michèle Lavagna, Roberto Bertacin

**Affiliations:** 1 Department of Aerospace Science and Technology, Politecnico di Milano, Via Giuseppe La Masa, 34, 20156 Milano, Italy; 2 Agenzia Spaziale Italiana, Via del Politecnico, 00133 Roma, Italy

**Keywords:** attitude determination, sensor architecture, nano-satellite, gyroscope, Sun sensor, magnetometer, GNSS receiver

## Abstract

The High Energy Rapid Modular Ensemble of Satellites (HERMES) is a constellation of 3U nano-satellites for high energy astrophysics. The HERMES nano-satellites’ components have been designed, verified, and tested to detect and localize energetic astrophysical transients, such as short gamma-ray bursts (GRBs), which are the electromagnetic counterparts of gravitational wave events, thanks to novel miniaturized detectors sensitive to X-rays and gamma-rays. The space segment is composed of a constellation of CubeSats in low-Earth orbit (LEO), ensuring an accurate transient localization in a field of view of several steradians exploiting the triangulation technique. To achieve this goal, guaranteeing a solid support to future multi-messenger astrophysics, HERMES shall determine its attitude and orbital states with stringent requirements. The scientific measurements bind the attitude knowledge within 1 deg ($1\sigma_{a}$) and the orbital position knowledge within 10 m ($1\sigma_{o}$). These performances shall be reached considering the mass, volume, power, and computation constraints of a 3U nano-satellite platform. Thus, an effective sensor architecture for full-attitude determination was developed for the HERMES nano-satellites. The paper describes the hardware typologies and specifications, the configuration on the spacecraft, and the software elements to process the sensors’ data to estimate the full-attitude and orbital states in such a complex nano-satellite mission. The aim of this study was to fully characterize the proposed sensor architecture, highlighting the available attitude and orbit determination performance and discussing the calibration and determination functions to be implemented on-board. The presented results derived from model-in-the-loop (MIL) and hardware-in-the-loop (HIL) verification and testing activities and can serve as useful resources and a benchmark for future nano-satellite missions.

## 1. Introduction

Multi-messenger astrophysics is a novel scientific field that uses information carried by various and diverse cosmic messengers, electromagnetic waves, neutrinos, and gravitational waves, to observe and understand astrophysical phenomena. In order to effectively collect the multiple sources, distributed space systems are becoming extremely popular and useful, either as primary detectors or as localization sentinels to subsequently alert other instruments and observatories. In all these cases, the benefits guaranteed by miniaturized spacecraft are evident. In fact, these small platforms allow an easier and cost-effective access to space, with a reduced time scale from design to operations and with a pragmatic possibility to implement distributed space missions. Nano-satellites have lowered by several orders of magnitude the difficulties and the costs to realize orbital constellations or multi-element space observatories. In this way, they have facilitated and have made more accessible astrophysical studies from space that require an observation baseline, such as interferometry or localization by triangulation.

The positive influence of miniaturized space platforms on the multi-messenger astrophysics field has been deeply exploited by the High Energy Rapid Modular Ensemble of Satellites (HERMES) mission [1]. HERMES is based on a constellation of nano-satellites in low-Earth orbit (LEO). The constellation is composed of 3U CubeSats, and it is built upon a twin project: the HERMES Technological Pathfinder (HERMES-TP), funded by the Italian Ministry for University and Research (MUR) and the Italian Space Agency (ASI); and the HERMES Scientific Pathfinder (HERMES-SP), funded by the European Union’s Horizon 2020 Research and Innovation Programme. Both the HERMES-TP and HERMES-SP projects provide three complete satellites to the constellation, for a total of six 3U CubeSats composing the space segment. The mission aims at the fast detection and localization of energetic astrophysical transients, such as gamma-ray bursts (GRBs), which are the electromagnetic counterparts of gravitational wave events, thanks to novel miniaturized detectors sensitive to X-rays and gamma-rays [2].

The astrophysical events’ localization has to be accomplished with an accuracy level ranging from a few degrees to arcminutes, as a function of the number of spacecraft composing the full HERMES constellation. This is achieved in a field of view of several steradians exploiting the triangulation technique [3]. In doing so, at least three spacecraft, separated by a minimum baseline of 1000 km, shall observe the same region of the sky by co-aligning the detectors’ lines of sight [4]. This is achieved by maneuvering the individual elements of the constellation to orient their fields of view in the desired directions with an autonomous attitude determination and control subsystem (ADCS) [5,6]. Then, any HERMES spacecraft shall estimate its full orbital and attitude states on-board. The first is needed to understand if the minimum baseline requirement is respected and to feed the environment models used in the attitude determination functions [7]. The latter is globally needed to have the ADCS properly working, achieving the expected mission goals. Specifically, the scientific requirements impose:

- A maximum attitude knowledge error of 1 deg at $1\sigma_{a}$;
- A maximum orbital position knowledge error of 10 m at $1\sigma_{o}$.

Therefore, despite the specific mission scenario analyzed, this research work can be applied and generalized to any spacecraft mission with similar attitude determination requirements, which are becoming common in complex and advanced nano-satellites.

However, this class of required performances is quite demanding for a nano-satellite because of its stringent mass, volume, power, and computation constraints. Moreover, the cost budget imposes a further difficulty to achieve the desired mission objectives. It is evident that the usage of extremely accurate sensors is typically not possible, and thus, an effective sensor architecture for full-attitude determination is needed. This paper discusses the one that was developed and verified for the HERMES satellites, but it is possible to state that the presented results are valid for CubeSat applications with the system constraints listed in Table 1.

Existing literature studies have addressed the problem of sensor architectures for spacecraft attitude determination in real missions for many years [8,9,10]. However, only in recent studies have the results been available for nano-satellites’ and CubeSats’ attitude determination subsystems. The work of Schmidt presented an attitude determination system based on Sun sensors, magnetometers, and gyroscopes to be processed by an extended Kalman filter (EKF) [11]. The simulations results proved errors in the order of 10 deg to 20 deg, which are acceptable for an experimental pico-satellite in-orbit testing mission, but they are not for missions with imposed scientific requirements. Similarly, Scholz presented real flight data from a pico-satellite mission that is capable of estimating the orbital position with an ∼1 × 10^2^ km accuracy [12]. A few years later, Springmann discussed an attitude determination system that utilizes rate gyros, magnetometers, coarse Sun sensors, and an EKF [13]. In this case, the attitude determination accuracy was about 2 deg to 3 deg in sunlight and decreased to about 7 deg to 8 deg when the states were estimated in eclipse. In the very last years, literature works about the ADCS in nano-satellites became more frequent, and they were often focused on improving the performance and the reliability of the developed system and sensor architectures. Byeon proposed a two-stage approach method for attitude determination that was robust to sensor faults [14]. In nominal conditions, this method is accurate in the order of 10^1^ arcsec, but it requires a star sensor, which may not respect the system constraints listed in Table 1. With a different approach, the work of Zhang exploited independent vector and gyroscope measurements to efficiently determine the spacecraft attitude [15]. This allowed reaching a high estimation accuracy, but it required a computational load and minimum sensor performances that are not suitable for nano-satellites. In these regards, the problem of attitude determination in nano-satellite was recently investigated by Fei [16], Li [17], and Ivanov [18]. These research works exploited miniaturized sensors; however, the obtained accuracy was in the range of 1 deg to 10^1^ deg, and they did not provide MIL or HIL test results. Experimental data were exploited in the work of Porras-Hermoso [19], who proposed a simple, yet reliable method to estimate the Sun-pointing direction in a micro-satellite mission. Exploiting solar panels and photodiodes, this work achieved an estimation accuracy in the order of 10 deg. Similar conclusions are also possible analyzing the mission results of the NetSat satellites, whose overview is summarized in the work of Scharnagl [20].

The effective sensor architecture proposed in this paper allows achieving a 1 deg attitude determination accuracy at $1\sigma_{a}$ in any section of the orbit, together with a 10 m orbital position estimation at $1\sigma_{o}$. To achieve these results, the sensor architecture is composed of tactical-grade micro-electro-mechanical system (MEMS) gyroscopes, quadrant diode fine Sun sensors, photodiodes for coarse Sun estimations, and magnetometers. The system is also equipped with a GNSS receiver for precise on-board orbit estimation. The attitude determination is based on the classical Quaternion ESTimation (QUEST) algorithm [21,22,23], which uses on-board calibrated sensor data. The orbit determination exploits GNSS measurements and an orbital propagator fused together in an EKF, which can be easily integrated with accelerometer measurements in the case of orbital control maneuvers.

### List of Contributions

The paper describes the sensor architecture to guarantee the attitude determination performance. It illustrates the sensor selection process, focusing on the hardware typologies and specifications. The available data were obtained from hardware-in-the-loop (HIL) functional testing, and the sensor performance is representative of real commercial off-the-shelf (COTS) components.

First, the discussion presents the sensor architecture, with a schematic description of the whole attitude and orbit determination block. This part also covers the sensor configuration on the spacecraft, highlighting the hot redundancies in the architecture, while the effectiveness and the efficiency of the proposed subsystem are defined and discussed. Furthermore, the methods and the software elements to fully determine the attitude and orbital states are illustrated and critically discussed with respect to the available sensor measurements.

Then, the sensor selection process is extensively described, and for each sensor in the proposed architecture, the main component’s specifications are reported and analyzed together with the primary test results. This part is structured according to the different sensor typologies, examining the performance of the gyroscopes, Sun sensors, magnetometers, GNSS receivers, and accelerometers.

Finally, an example attitude determination performance is reported and discussed with respect to the sensor performance and specifications. In particular, the direct influence of the sensor architecture on the final output of the determination functions is highlighted. In this part, the presented results were derived from model-in-the-loop (MIL) with hardware calibrated simulations.

The aim of this study was to fully characterize the proposed sensor architecture, with respect to the available attitude and orbit determination performance. This was achieved thanks to an extensive analysis of the sensor test results, with particular attention on the critic performance for each sensor family and thanks to an end-to-end verification of the final attitude and orbit estimation accuracy. All the presented results were derived from HIL and MIL verification and testing activities, and they can serve as useful resources and a benchmark for future nano-satellite missions.

## 2. Effective Sensor Architecture

The effective sensor architecture for full-attitude determination in the HERMES nano-satellites was developed starting from the imposed mission requirements and system constraints. In addition to those described in the previous section, the system is required to determine its full orbital and attitude states in any point of the orbit, and it shall be capable of withstanding failures in the hardware components. For these reasons, the sensor architecture was selected to be operative both in sunlight and in eclipse and with a few degrees of hot redundancy.

First, the mass, power, and cost limitations indicated discarding the star sensors as the primary attitude determination sensors. The possibility to host this sensor typology in a redundant configuration is extremely remote for a nano-satellite. Moreover, the attitude determination accuracy requirement is not extremely strict to force the design to use star sensors. Then, since the attitude state estimation shall not be dependent on the relative attitude with respect to the Earth, the primary attitude sensors were selected to be Sun sensors and magnetometers.

The latter are also needed to actuate magnetic torquers, which are very common in nano-satellites. The maximum attitude estimation error drove the selection of fine Sun sensors with maximum measurement errors in the order of ∼1 deg. However, to avoid the presence of too many fine sensors for redundancy purposes, the sensor architecture was based on a combination of fine Sun sensors in the Sun’s primary axes and of distributed photodiodes to retrieve a coarse Sun direction. Note that the photodiodes shall also guarantee the detection of the Sun with a full-sphere field of view, with no blind spots.

The requirement to determine the full-attitude state in any section of the orbit imposed the inclusion of gyroscopes for dead-reckoning state propagation while in eclipse. The gyroscope measurements shall be continuously calibrated on-board in order to minimize attitude drifts due to the gyroscope’s bias. Analogously, to keep the desired performance, the magnetometers and the Sun sensors are also calibrated with on-board techniques. Specifically, the magnetic sensor’s measurements shall be corrected for bias and scale factor errors, while the fine Sun sensors’ measurements are adjusted for the presence of Earth’s albedo.

This sensor architecture requires also the estimation of the orbital position state. This is needed to feed the reference environment models for full-attitude determination and for sensors’ calibration. Moreover, this is needed in guidance functions, which need to compute reference attitude states relative to the Earth. Finally, it is fundamental to achieve the mission objectives with the imposed accuracy requirement also on the orbit determination. In the presented architecture, the primary sensor is a GNSS receiver. For redundancy purposes, the GNSS measurements are combined in an EKF with an on-board SGP4 orbital propagator [24] and with the accelerometers’ measurements. The former can be updated with a two line element (TLE) set received from the ground. The latter were included because the accelerometers are typically embedded in MEMS inertial measurement units (IMUs) hosting gyroscopes and magnetometers, and they also enable the possibility to deal with orbital control maneuvers. In any case, the measured non-gravitational perturbation accelerations, despite being very disturbed due to the noise level of typical tactical-grade MEMS accelerometers, allow slightly improving the GNSS-based orbit determination accuracy, and they allow increasing the reliability of the on-board orbital propagation [7].

**Figure 1:**
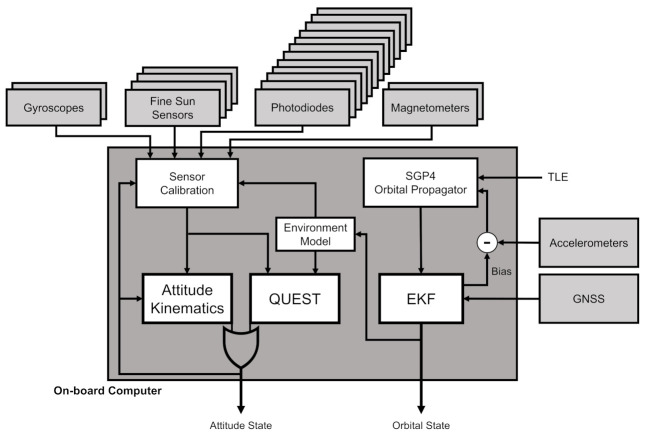
Sensor architecture scheme.

The overall sensor architecture is reported in Figure 1 together with the attitude determination blocks. In the scheme, the interfaces between the different elements and the hot redundancies are graphically reported. In particular, the gyroscopes, the magnetometers, and the Sun sensors have one degree of redundancy, each having on-board 2 three-axis gyroscopes, 4 fine Sun sensors, 12 photodiodes, and 2 magnetometers. The GNSS receiver has no redundant components, its failure being counteracted with the support of the three-axis accelerometer together with the SGP4 orbital propagation.

The sensor configuration further maximizes the efficiency of the sensor architecture, which is defined as the number of sensors per given attitude determination functionality. The fine Sun sensors are aligned with the four body axes contained in the plane orthogonal to the solar panel’s wings, in a way that, during the nominal attitude modes, at least one fine sensor is used. These are not installed on the surfaces not containing solar cells, where the Sun direction is detected with the coarse photodiode sensors. In this way, each of the four primary Sun directions has one main fine sensor and two redundant coarse photodiodes, while the remaining two directions have only the two redundant coarse photodiodes. Globally, the twelve photodiodes cover the full-sphere field of view around the spacecraft and act as redundant coarse Sun sensors (CSSs) in case one of the primary fine sensors fails.

**Figure 2:**
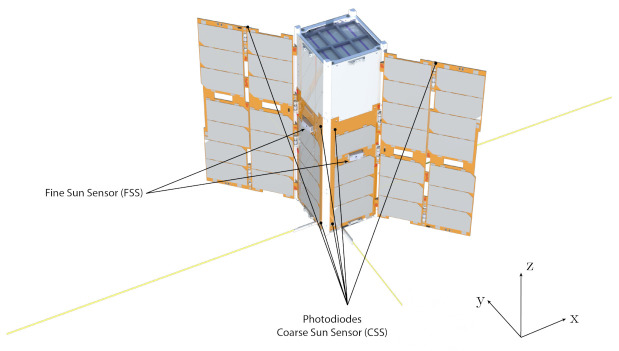
Spacecraft configuration.

**Figure 3:**
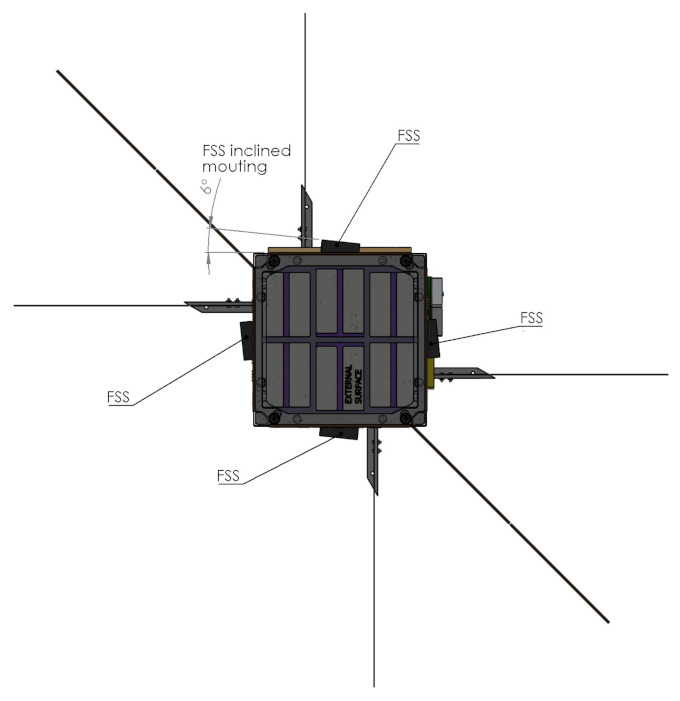
Fine sun sensors’ mounting.

Figure 2 shows the spacecraft configuration and highlights the position of the Sun sensors for the three illustrated surfaces. The photodiodes placed on the solar wings’ edges are those aligned with the *z* axis, while those on the side panels are aligned with the −x and −y axes. The configuration is specular for the remaining six photodiodes on the other three faces. The fine Sun sensors (FSSs) are mounted on the side panels to be aligned with the four primary Sun directions. In reality, as shown in Figure 3, they are inclined 6 deg with respect to these axes in order to have the entire field of view clear from interferences with the solar wings.

The other sensors are configured in a way to minimize disturbances and external interferences. Namely, the magnetometers are mounted as far as possible from the magnetic torquers and from the electronic boards, while the gyroscopes and the accelerometers are placed far from the sources of vibrations (e.g., rotating devices).

### Full-Attitude Determination with Sensor Measurements

The proposed sensor architecture guarantees a continuous availability of measurements to estimate the attitude states. In fact, also in case of the failure of some sensors, the available redundant components can be exploited to continue with the nominal operations. The attitude determination section’s algorithms use the calibrated Sun and magnetic sensor measurements:

(1) $${\hat{s}}_{C},\;b_{C},$$

to statistically solve Wahba’s problem with the QUEST implementation for quaternions [23]. The calibrated data were obtained from the raw measured ones as

(2) $${\hat{s}}_{C}={\hat{s}}_{M}-{\hat{s}}_{E}$$

(3) $$b_{C}=\left(I_{3\times 3}+D_{b}\right)b_{M}-\varepsilon_{b_{b}},$$

where $b_{M}$ and ${\hat{s}}_{M}$ are, respectively, the measured geomagnetic field and Sun direction, $\varepsilon_{b_{b}}$ is the estimated bias of the magnetometer, $D_{b}$ is the fully populated matrix of scale factors and non-orthogonality magnetometer errors, and ${\hat{s}}_{E}$ is the estimated error in the Sun direction due to the influence of the Earth’s albedo. The magnetic calibration parameters are continuously estimated on-board with a sequential centered iterative algorithm [25]. The Sun sensors’ correction for the albedo are computed knowing the position of the Earth and its irradiance and reflection models [26], the spectral response of the photodiodes, and the measurement functions of the Sun sensors. These algorithms are valid in sunlight, where it is assumed to exploit the Sun and magnetic field measurements, with the addition of the position vector estimate by the orbit determination algorithms, r, and the current time, *t*. These last two quantities are used in the environment models in order to compute the geomagnetic field and the Sun direction in the inertial reference frame: $b^{I}$ and ${\hat{s}}^{I}$. The environment models are based on the Chebyshev ephemerides of the Sun and on the IGRF model of the Earth’s magnetic field [27]. Moreover, the spacecraft position vector is also used to compute the Earth parameters used in the albedo correction functions.

Whenever two independent vector measurements from the sensors are not available (e.g., during eclipses), a dead-reckoning attitude propagation is initialized from the last valid attitude state. The attitude kinematics is then propagated with the unbiased output of the gyroscopes. The gyroscope calibration function estimates the angular velocity biases, $\varepsilon_{b_{\omega }}$, with a sequential complementary filter [28], whose bias dynamics is expressed as follows:

(4) $${\dot{\varepsilon }}_{b_{\omega }}=-k_{b_{\omega }}\mathrm{sign}\left({\tilde{q}}_{4}\right){\tilde{q}}_{1:3},$$

where $\tilde{q}=\left[{\tilde{q}}_{1:3},{\tilde{q}}_{4}\right]$ is the estimated attitude quaternion with its vector and scalar parts. The angular velocity is then corrected with the current best estimate of the biases:

(5) $$\omega_{C}=\omega_{M}-\varepsilon_{b_{\omega }},$$

where $\omega_{M}$ is the spacecraft’s measured angular rate vector. Note that the sensor calibration is not updated during the dead-reckoning propagation periods.

The static attitude determination logic uses the calibrated sensor measurements to implement the QUEST algorithm. The magnetometer and Sun sensors are weighted in this statistical implementation according to their quality, defined as the mean of the measurement’s variance computed in the sensor postprocessing functions:

(6) $$a_{b}=\frac{1}{\frac{1}{S}\sum_{i=1}^{S}\sigma_{b}\left(t_{S+1-i}\right)},\;\mathrm{and}\;a_{\hat{s}}=\frac{1}{\frac{1}{S}\sum_{i=1}^{S}\sigma_{\hat{s}}\left(t_{S+1-i}\right)},$$

where $a_{b}$ and $a_{\hat{s}}$ are non-negative weights respecting $a_{b}+a_{\hat{s}}=1$. The outcome of the statistical attitude determination is the best estimation of the spacecraft’s orientation, in terms of the quaternion, $\tilde{q}$, and angular velocity, $\tilde{\omega }$. The dynamic attitude determination complementary filter uses the output of this static attitude determination section, combined with the angular rates’ measurements, to obtain the updated bias values, as described before. At any time of the mission, the output of the full-attitude determination functions is the best estimate of the attitude state vector.

The orbit determination algorithm is based on a loosely coupled GNSS and inertial navigation system (INS) integration. Namely, the accelerometers and the SGP4 orbital propagator are exploited to update the orbital state vector generating the INS preliminary estimate, which is then compared with the GNSS measurements to calculate the measurement error. This measurement error signal is fed to an extended Kalman filter (EKF) [29], which outputs an updated estimate of the position and velocity errors, as well as the accelerometer bias term estimate. Eventually, the output of the EKF is used to correct the INS preliminary estimate, whereas the bias term is fed back to the integration block of the accelerometer measurements. The orbit determination section’s algorithms provide a full orbital state estimate in terms of inertial position, r, and velocity, v, using positioning and acceleration measurements:

(7) $$r_{M},\;v_{M},\;a_{M}.$$

Note that the accelerometer bias term estimate is continuously updated to calibrate the accelerometer measurements:

(8) $$a_{C}=a_{M}-\varepsilon_{b_{a}}.$$

Whenever the GNSS is experiencing an outage period or a fault, the on-board SGP4 orbit propagator is used as the synthetic measurement element for the INS input. In this case, the EKF exploits a different measurement model and covariance matrix with respect to the nominal mode with GNSS and accelerometer coupling. Obviously, the approximate orbital model introduces propagation errors, and an inherent position drift cannot be avoided. However, the coupling with the EKF helps in accounting for the orbital error dynamics, and it determines a slower divergence rate with respect to a pure on-board orbital propagation including the same perturbation terms.

## 3. Sensor Selection

The sensor selection process was carried out on a pool of COTS sensor components respecting the mass, volume, and power constraints for nano-satellites. Among these elements, the available performance was critically assessed also considering the cost limitations and the electrical and data interfaces’ compatibility. Note that the requirements for COTS components came from the cost budget’s limitations and from the philosophy driving nano-satellite missions. In this section, the selected sensors are presented with the details of the critical performance and of the analyses that led to their choice.

### 3.1. Gyroscopes

Gyroscopes are needed to guarantee good propagation performance during the eclipse period, and they also provide continuous data for feedback action on the desired reference velocity. Given the dimensions of nano-satellites, MEMS gyroscopes are mandatory and shall be compliant with the power budget. The proposed architecture requires two redundant gyroscopes, with accuracy levels of tactical-grade sensors. In fact, the eclipse propagation of ∼30 min shall not accumulate more than 3 deg of error to comply with the maximum $3\sigma_{a}$ error requirement. Taking into account the on-board calibration, the parameter to focus on is the bias instability of the gyroscope. Thus, the sensor selection focused on components with a maximum bias instability of 5 deg/h, which are fully compliant with the tactical-grade limits [27]. Moreover, the mass and volume constraints suggest searching for a combined IMU with three-axis gyroscopes, magnetometers, and accelerometers.

**Figure 4:**
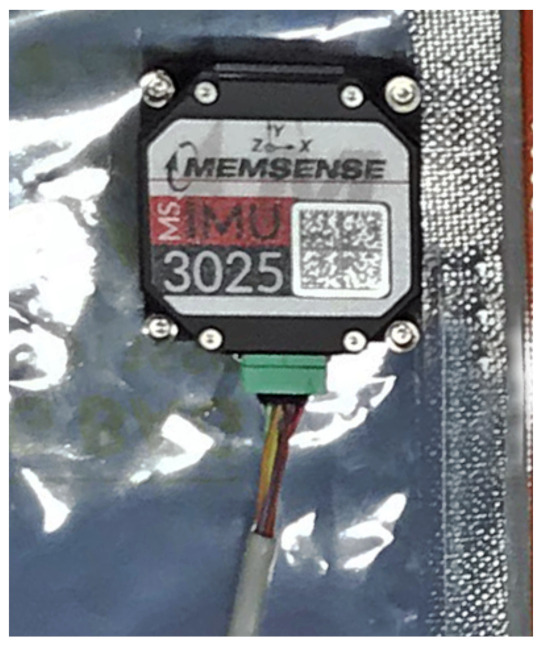
Memsense MS3025M IMU.

**Figure 5:**
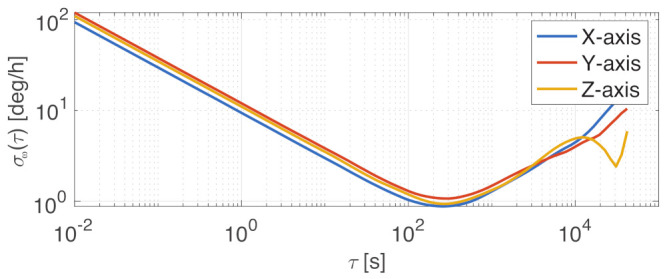
Allan variance, $\sigma_{\omega }\left(\tau \right)$, for Memsense MS3025M gyroscope.

The selection identified the Memsense MS3025M IMU as a good choice as primary gyroscope for this sensor architecture. This component provides about 1 deg/h of gyro bias instability and sub-seven micro-g accelerometer bias instability. This is performed with less than 18 g of mass and 1 W of power. The package dimensions are fully compatible with nano-satellite specifications, the size of the sensor being 28×28×11.5mm. The sensor component under testing is shown in Figure 4, and the measured performance is reported in the specification matrix in Table 2. The gyroscope’s stochastic processes’ analysis was performed with the Allan variance method, whose results are shown in Figure 5. The noise performance test was performed sampling the zero-rate outputs of the IMU at a 25 Hz sampling rate through the RS-422 serial interface. The IMU was configured with a full-scale range of 75 deg/s, and the steady-state component temperature was at 38 ${}^{\circ }C$. The Allan variance data were sampled over a time span of 6 h.

**Figure 6:**
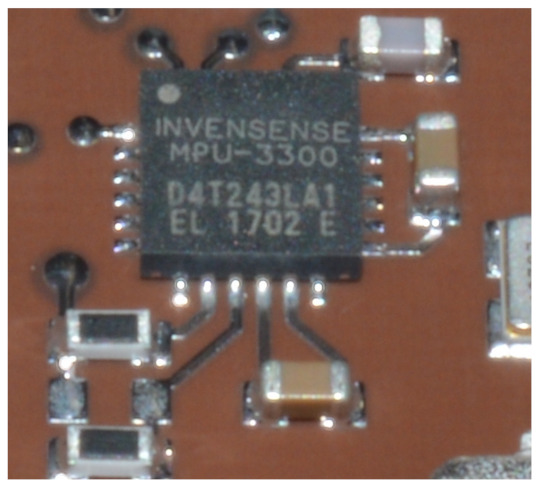
Invensense MPU3300 gyroscope.

The hot redundancy with an identical MS3025M component is not possible in the HERMES spacecraft, and in general, it is not easy to accomplish with the imposed system constraints. Thus, gyroscope hot redundancy was performed with another MEMS gyroscope with a lower mass and consumed power. In this case, the same performance cannot be achieved, but good industrial-grade gyroscopes are available as qualified COTS components. The Invensense MPU3300 three-axis gyroscope provides around 5 deg/h of bias instability in a mass of less than 1 g, consuming ∼12 mW and with dimensions of 4×4×0.9mm. In this case also, the component performance is listed in Table 3, stating that it was tested sampling the component at 25 Hz through an I2C interface. The component temperature is not directly available, but the room temperature during the test was at 23 ${}^{\circ }C$. Figure 6 shows the gyroscope integrated into one of the spacecraft’s electronic boards.

It can be noted that, despite the lower accuracy grade, this component has good performance and allows maintaining the imposed requirements, even in the case of a failure of the primary gyroscope. The low bias instability allows maximum propagation errors along the eclipse periods lower than 3 deg at $3\sigma_{a}$. Moreover, the low noise level of this industrial-grade miniaturized component makes it an excellent choice as a secondary redundant gyroscope. The selection of the gyroscope data to use, either the primary or the secondary, depends on a quality index of the raw measurements, which can be computed as in [7].

### 3.2. Sun Sensors

Sun sensors are electronic devices capable of measuring the direction of the incoming sunlight in the body reference frame. Comparing this measurement with the known position of the Sun in the inertial reference frame allows solving the attitude problem, given another independent vector direction is measured as well. As said, in the proposed sensor architecture, the geomagnetic field vector completes the measured attitude directions.

Miniaturized Sun sensors for nano-satellite applications are commonly based on photodiodes. These can be utilized individually as independent Sun angle sensors or arranged in groups to compose more refined Sun direction sensors. In the first case, the complete three-dimensional direction of the Sun is only available placing at least three photodiodes aligned with different body axes. In the second case, a single sensor component can directly measure the Sun unit vector in the body frame. The proposed sensor architecture utilizes both alternatives for the fine and the coarse Sun measurements.

#### 3.2.1. Fine Sun Sensors

The fine Sun sensors selected for the presented nano-satellite application are based on quadrant photodiode technology, which is capable of measuring the Sun vector direction by detecting the differential signals on the quadrant that are generated by the sunlight passing through a hole in the sensor’s casing. Then, the signals are sampled, filtered, and processed with an analog-to-digital converter (ADC) integrated with a micro-controller. The measurements are finally made available for the attitude determination functions through an I2C communication interface. In the HERMES nano-satellites, the selected COTS sensors with qualified space operations are produced by GOMSpace and commercialized as the NanoSense fine Sun sensors (FSSs).

Each Sun sensor has a theoretical field of view of 60 deg at a half angle. Thus, each sensor covers a solid angle of πsr, and six sensors can cover the full sphere around the spacecraft. However, for many nano-satellites such as HERMES, the Sun direction will be contained in the spherical segment facing the solar wings and a lower number of fine sensors can be employed. Indeed, in HERMES, four FSSs are mounted on the faces aligned with the ±x and ±y axes, with reference to Figure 2. Despite their inclined mounting angle of 6 deg shown in Figure 3, they cover the complete spherical segment of half angle 60 deg cut by two planes parallel to the x−y plane. With this configuration, at least one Sun sensor has the Sun in view during nominal operations. With a random attitude, the Sun direction is contained in the covered spherical segment with a probability of ∼85%. The remaining conditions are fully supported by the presence of the photodiode coarse Sun sensors.

**Figure 7:**
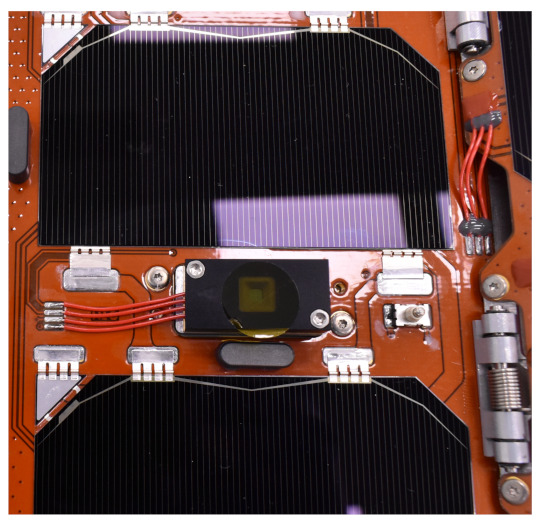
GomSpace FSS sun sensor integrated on the solar array. Note the Kapton layer to protect the sensor’s hole. Photo courtesy of DHV Technology.

The inclined mounting angle allows having no portion of the FSS’s field of view obstructed by the solar wings, and it reduces the incidence angle of the Sun with respect to the sensor’s line of sight when the Sun is perpendicular to the solar arrays. Indeed, when the Solar arrays generate their maximum power, the Sun has an incidence angle with respect to the sensor of 39 deg. The best performance would have been available with the Sun directly perpendicular over the sensor, but in nominal conditions, this would have required mounting the sensor on the solar wing. However, the flexibility and the oscillations of the deployable surface would have reduced the determination accuracy of the sensor architecture. Then, the FSSs were solidly attached to the body-mounted solar panels, as shown in Figure 7.

**Figure 8:**
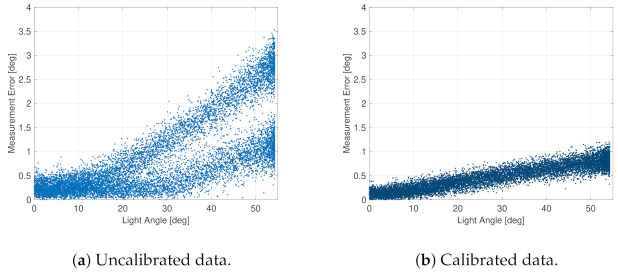
Light measurements with the FSS.

In this configuration, with the nominal Sun incidence angle in the range from ∼35 deg to 45 deg, the mean calibrated measurement error was in the order of 0.5 deg in the worst conditions around 1 deg. These values were retrieved by testing the fine Sun sensor with variable incident light, as shown in Figure 8. This clearly shows the performance of the sensors, which were sampled at 10 Hz with an artificial light source in dark laboratory conditions. These results were obtained by sweeping a light source for an arc of 120 deg from the positive to the negative direction. Then, the raw data measurements were converted into the Sun angle, geometrically calibrated, and directly compared with the reference values. The geometrical calibration exploited a look-up table filled with 81 calibration points associated with the azimuth and elevation of the light source with respect to the sensor’s focal plane and line of sight. The calibration points were evenly distributed both in azimuth and elevation to cover the entire sensor’s field of view. When both direction angles of the incoming light source were measured from the sensor, they were corrected in the postprocessing functions, subtracting the known error angles, which were retrieved by the linear interpolation of the nearest points in the calibration table. The effectiveness of the calibration procedure is evident comparing the uncalibrated data, in Figure 8a, with the calibrated ones, in Figure 8b. In particular, this operation reduced the scale factor errors and removed the geometrical distortion due to the non-orthogonality of the sensor assembly. Looking at the uncalibrated raw data, the measurement error had two distinct trends. They were due to the asymmetry of the component with respect to different directions of the incoming light. In this specific case, the positive angle direction had a lower scale factor error than the negative one. This geometrical distortion was completely removed in the calibrated measurements, where only a slight scale factor error was still present. Note that the calibration procedure also improved the measurement accuracy at zero angle, when the light source is directly above the sensor, because the calibration table accounts for the geometric biases as well. Table 4 summarizes the main performance results retrieved from the testing activities.

#### 3.2.2. Photodiodes and Coarse Sun Sensor

Complete Sun detection coverage is achieved with the usage of photodiodes configured to cover all the satellite’s faces, in a way that composes a coarse Sun sensor (CSS). In fact, a single photodiode is a simple Sun angle sensor, whose output is an electric current proportional to the cosine of the incident sunlight’s angle, α, as

(9) $$I=I_{0}\cos\left(\alpha \right),$$

where $I_{0}$ is the maximum current produced when the Sun is directly above the photodiode. In the proposed sensor architecture, each of the six spacecraft faces is equipped with two hot redundant identical photodiodes. Their individual measurements are averaged, unless a failure is detected in one of the two components, as described in [7]. Then, for each face, a single current value is available, and it can be associated with a Sun angle with respect to that face:

(10) $$\alpha_{n}=\cos^{-1}\left(\frac{\mathrm{mean}(I_{1_{n}},I_{2_{n}})}{I_{0}}\right),$$

where *n* denotes the normal direction versor of each face in the body reference frame, ${\hat{n}}_{n}$. The complete Sun direction can be computed from Equation (9) as:

(11) $${\hat{s}}_{i}^{B}=\frac{s_{i}^{B}}{|s_{i}^{B}|},\;\mathrm{and}\;s_{i}^{B}=\sum_{n=1}^{6}\cos\left(\alpha_{n}\right){\hat{n}}_{n},$$

where ${\hat{s}}_{i}^{B}$ is the Sun direction in the body reference frame.

The important performance for each single photodiode is the accuracy with respect to the ideal cosine law, the stability of the maximum current value, and the spectral response with respect to the incoming light sources. The latter is very important to implement proper albedo-filtering functions because, knowing the position of the Earth, it allows estimating the current intensity due to the Earth’s radiation. The HERMES nano-satellites use the Osram SFH 2716 A01 photodiodes. They have a spectral sensitivity very similar to the human eye, with low influence from infrared radiation, which is positive for reducing the influence of the Earth’s irradiance. Moreover, they have a very small surface-mounted package, with an extremely low weight of 4 mg.

**Figure 9:**
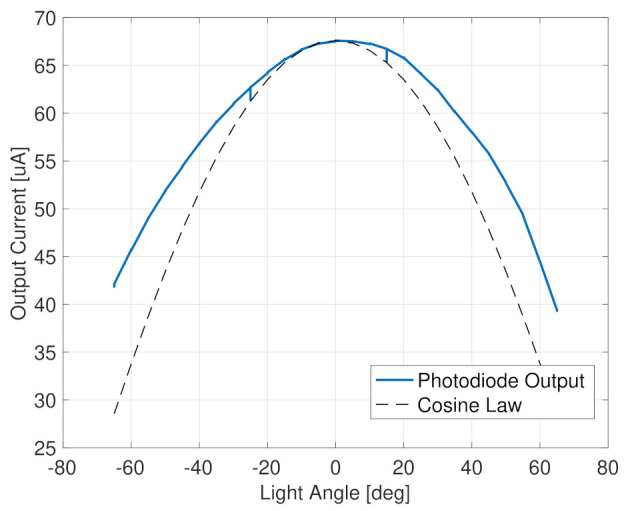
Light measurements with CSS photodiode.

The directional response is smooth and symmetric, allowing an easy calibration with respect to the cosine law, as is evident from the test results reported in Figure 9. These data were acquired with a sampling rate of 20 Hz, exploiting the ADC associated with each photodiode and the microcontroller that manages all these 12 light sensing elements on the spacecraft. In this case also, the test is performed by rotating a known light source along an arc of 120 deg. Calibrating the incoming light intensity with respect to the Sun intensity in LEO, the maximum output current was in the order of 67 μA, and the error with respect to the cosine law can be parametrized with a single coefficient as:

(12) $$I=I_{0}\cos\left(k\alpha \right).$$

In the proposed example case, the calibration parameter results were approximately equal to k≃0.8. The calibrated formula in Equation (12) shall be used in place of the one in Equation (9). Then, Equations (10) and (11) shall be modified accordingly, if the photodiode calibration needs to be applied.

### 3.3. Magnetometers

Magnetometers are the other sensors that are needed to fully determine the attitude states of the spacecraft. In fact, at least two independent vector measurements are needed to estimate the full-attitude state. Moreover, the geomagnetic field knowledge is often useful on-board to drive magnetic actuators or to manage the spacecraft’s magnetic dipole. For the nano-satellite application, MEMS magnetometers are commonly employed in place of classical fluxgate components. Despite the former being less accurate with respect to the latter, these sensors are extremely small, light, and power-efficient, and the accuracy level is in the order of 1 deg, which is compliant with the imposed requirements.

**Figure 10:**
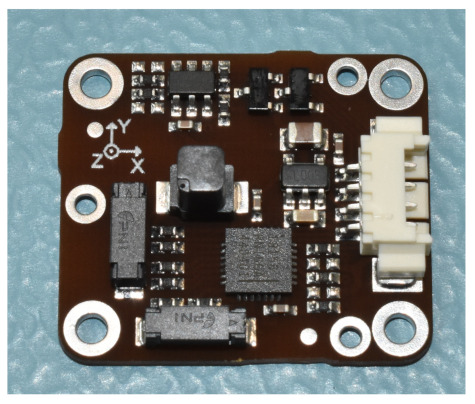
PNI RM3100 magnetometer. Photo courtesy of GOMSpace.

**Figure 11:**
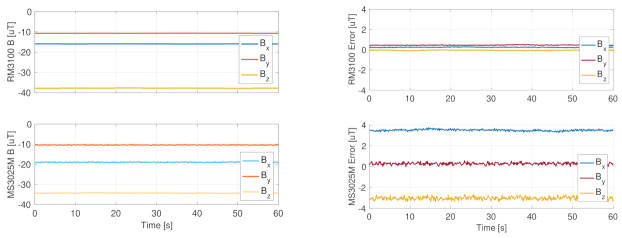
RM3100 and MS3025M magnetometer measurements.

The proposed architecture requires two redundant magnetometers, which were selected looking for the noise level performance. In fact, the biases, the non-orthogonality, and the scale factor errors can be corrected with the on-board calibration algorithm described before. In these regards, a lower noise in the magnetic measurements would result in better performance of the calibration functions and, in general, in improved quality of the whole attitude determination and control subsystem. The selection identified the PNI RM3100 geomagnetic sensor as a good candidate for primary magnetic measurements with its military-grade performance. In fact, this sensor, shown in Figure 10, is based on magneto-inductive MEMS sensor technology, which provides high resolution, low power consumption, no hysteresis, and low noise, if compared to other miniaturized components. Its noise level is around 15 nT, which is at a maximum 0.075% of the measured geomagnetic field value in LEO. Moreover, this value is in the order of the sensitivity of the instruments, that is 13 nT. Thus, the magnetic measurements are extremely clean with this component, as is evident from the uncalibrated results reported in Figure 11, which were sampled at 10 Hz through an I2C data interface in laboratory environment conditions. Table 5 reports the most-relevant specifications of the PNI magnetometer retrieved from the testing activities. This magnetic sensor is commercially available with an integrated temperature sensor and an I2C digital interface from GOMSpace with the product name NanoSense M315.

The secondary magnetometer is embedded in the Memsense MS3025M IMU, since the effort of minimizing the mass, volume, and power of the overall sensor architecture suggested the selection of a nine-axis IMU component. The choice of this sensor was mainly driven by the gyroscope performance, but the quality of the magnetic measurements is anyway good for a secondary redundant component. From the uncalibrated results presented in Figure 11, it is evident how the secondary magnetometer has a greater noise level and biases, but it allows in any case achieving the desired attitude determination goals. The reported measurements were sampled in parallel with those of the PNI magnetometer at the same sampling rate. Moreover, note that Figure 11 reports the raw measurements, without any postprocessing calibration applied. In this case also, the most-important specifications are listed in Table 6.

### 3.4. GNSS Receiver

**Figure 12:**
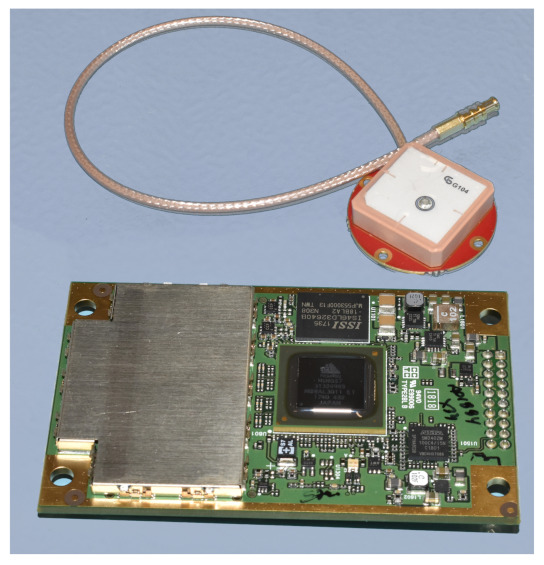
Novatel OEM719 GNSS module and Tallysman TW1322 antenna.

The presence of an on-board GNSS receiver allows implementing autonomous orbit determination functions, which are fundamental to have an accurate full-attitude determination subsystem. Moreover, in the HERMES nano-satellites, the position estimation requirements are anyway very stringent. In the proposed architecture, the selected GNSS module was the Novatel OEM719 because of its proven flight heritage and its compatibility with some existing nano-satellite interface electronic boards. The module can be associated with different antennae, but the Tallysman TW1322 was used in this case. Both elements are shown in Figure 12.

The GNSS receiver testing is not really representative of the space operation conditions, because of the different altitudes and velocities in place. Indeed, the module shall be applied with the CoCom limits removed for in-orbit applications. Thus, the testing activities were limited to verify the correct functionality of the antenna and of the receiver, solving for a ground-based position and velocity GNSS fix. Yet, the orbit determination performance was not assessed. Table 7 lists the main specifications of the GNSS sensor.

### 3.5. Accelerometers

Accelerometers are used to measure the on-board non-gravitational accelerations, such as those generated by non-gravitational external perturbations and orbital control maneuvers. The selection of the Memsense MS3025 IMU guarantees the availability of a three-axis accelerometer. This sensor’s measurements allow marginally improving the GNSS-based orbit determination accuracy and increasing a little the reliability of the on-board orbital propagation [7]. Indeed, the noise level is in the order of ∼1 μg at $1\sigma_{g}$, which is in the same order of the main non-gravitational external perturbations. However, it can be very useful if the proposed sensor architecture is applied to a platform with orbital control capabilities.

**Figure 13:**
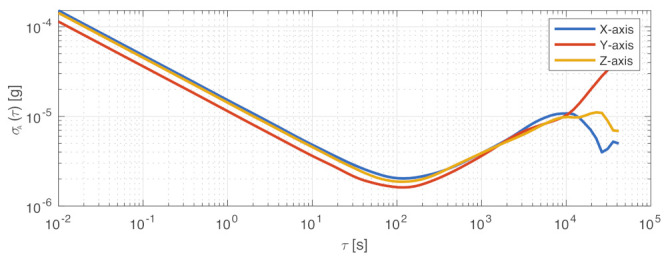
Allan variance, $\sigma_{A}\left(\tau \right)$, for Memsense MS3025M accelerometer.

The measured performance is reported in the specification matrix in Table 8. In this case also, the Allan variance method was used to perform the accelerometer’s stochastic processes analysis, whose results are shown in Figure 13. The zero-rate outputs of the IMU’s accelerometer were sampled at 25 Hz through the RS-422 serial interface. The IMU was configured with a full-scale range of 2 g, and the steady-state component temperature was at 38 ${}^{\circ }C$. The Allan variance data were sampled over a time span of 6 h.

## 4. Full-Attitude Determination Performance

The full-attitude determination accuracy is strongly dependent on the sensor performance. In fact, the quality of the available measurements sets a physical limit to the lowest determination error that can be achieved. For these reasons, a credible attitude determination design has to be founded on HIL testing results with the available sensors. Then, even if the attitude determination functions are going to be verified with MIL tests, or software-/processor-in-the-loop (SIL/PIL) analyses, the supporting system and dynamical simulators shall be calibrated with respect to real hardware performance.

The attitude determination performance was verified by means of numerical simulations composing a model-in-the-loop testing campaign, exploiting a functional engineering simulator (FES) developed for small satellite applications in Earth orbits. The HERMES nano-satellite dynamics accounts for realistic system characteristics, such as the measured inertia properties and dimensions. The nominal orbital environment is described according to international standards [30] accounting for the nominal orbit of the spacecraft: an equatorial LEO with an altitude of 550 km. The simulation was run for four orbital periods, starting from a random attitude state. The nominal attitude mode was a nadir pointing mode, which is ideally tracked by an ideal controller (i.e., the pointing error was 0 deg). The FES contains detailed functional models of all the sensors composing the discussed architecture, whose parameters and performance were those discussed in the previous sections, retrieved from HIL testing activities. The full-attitude determination functions were executed at 10 Hz, and the sensor models were sampled at the same frequencies as the real hardware components. The output results were analyzed in terms of three-axis attitude determination error angle and three-axis orbit determination error position. The FES runs of MATLAB/Simulink with a fixed step numerical integrator with a sample time of 0.01 s. The numerical calculations of the attitude and orbit determination functions were executed with 32 bit floating numbers, to be representative of the numerical computations to be executed by the on-board processor. Table 9 summarizes the main simulation parameters used in the presented analyses.

The attitude determination performance was assessed in several pointing modes, with random initial states and with realistic control performance. Moreover, the closed-loop determination and control functions were also verified together. However, to show and present the available attitude determination accuracy, an ideal attitude pointing control was exploited. In this condition, the attitude states, initialized with a random condition, were quickly aligned with the nominal operative nadir pointing. Then, along the orbit, the ideal controller made two yaw rotations in order to maintain the solar arrays’ fronts face the Sun. In this way, it was possible to study the attitude determination functions independently of the controller, and the resulting performance was only representative of the determination block. The proposed results were anyway significant and in line with the realistic results available for the entire HERMES mission.

**Figure 14:**
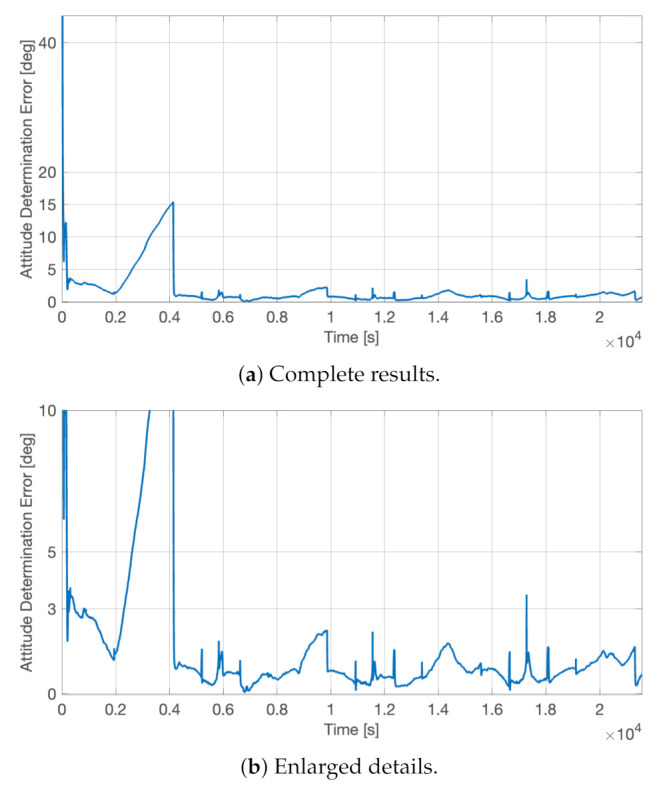
Full-attitude determination error, $\alpha_{err}$, in nadir-aligned attitude state.

The three-axis attitude determination error, $\alpha_{err}$, satisfied the imposed requirements, as shown in Figure 14. This performance evaluation parameter is defined as the angle between the desired pointing direction, ${\hat{v}}_{target}$, and the actual one, $\hat{v}$, as

(13) $$\alpha_{err}=\cos^{-1}\left(\hat{v}\;\cdot \;{\hat{v}}_{target}\right).$$

This figure of merit is used in HERMES since the scientific requirement is formulated on the three-axis overall error angle in estimating the attitude state. Thus, it is not relevant to understand how this error is split into the classical roll, pith, and yaw components. As soon as the system was initialized, the estimated attitude states converged to the real ones with a determination error that was below 1 deg at $1\sigma_{a}$ whenever the on-board determination functions were fully calibrated. Indeed, the attitude determination error stayed below 3 deg for the entire time, as visible in Figure 14b, except for an instantaneous peak at *t* ∼ 1.7 × 10^4^ s and for the increasing error between 2000 s and 4000 s. The former was only a slightly larger jump in the determination functions, similar to the others that are commonly present during the attitude estimation process. These small peaks in the attitude determination error are typically due to a switch in the fine Sun sensor with the Sun currently in view. This sensor change happens during the yaw rotations and for attitude conditions in which the Sun is at the border of the field of view of one sensor. For instance, the same peaks would not be present in the case in which the attitude determination is executed in inertial attitude mode, whereas the initial attitude determination error rise was associated with the first dead-reckoning period during the first eclipse, when the gyroscope’s biases were still not completely estimated. Thus, along the first orbit, the attitude determination system was not fully calibrated yet. This small issue can be partially solved by initializing the calibration functions with ground-estimated biases, even if the launch event will for sure wreck the calibration values. In this regard and to minimize the risk of possible counter calibrations, the HERMES calibration functions were initialized with zero biases. However, whenever the biases were correctly estimated on-board, the dead-reckoning attitude propagation did not go over the $3\sigma_{a}$ error threshold. This is visible in Figure 14b for the following three eclipse periods at times *t* ∼ 0.9 × 10^4^ s, *t* ∼ 1.4 × 10^4^ s, and *t* ∼ 2.0 × 10^4^ s.

**Figure 15:**
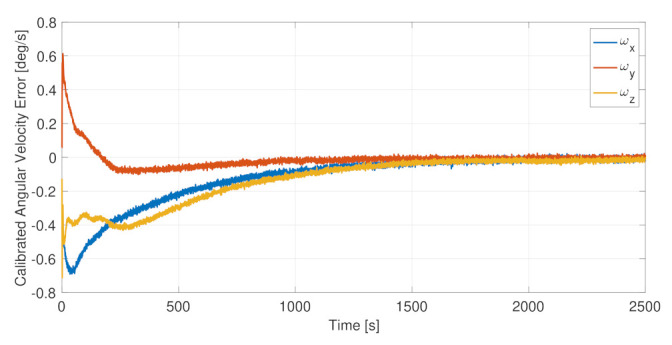
Angular velocity calibration error.

**Figure 16:**
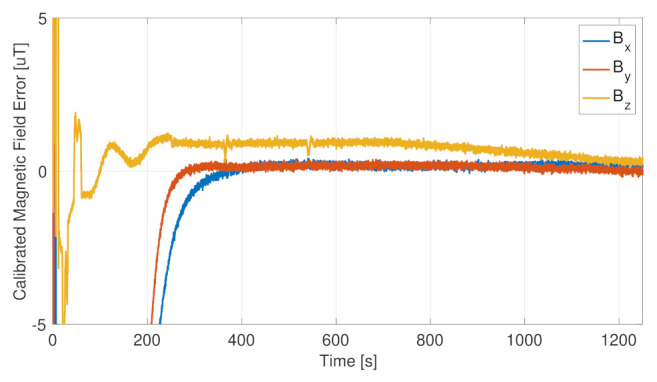
Magnetic field calibration error.

The sensor calibration was beneficial for the full-attitude determination functions, improving the estimation accuracy both in sunlight and in eclipse. The on-board autonomous calibration is continuously running, being able to calibrate the drifts in the sensor errors and to recalibrate the whole system, if the primary sensor fails and a redundant one is set in use. This is possible thanks to the relatively fast dynamics of the calibration algorithms. Figure 15 and Figure 16, respectively, report the residual error in the calibrated angular velocities and in the calibrated magnetic field. In both cases, the calibration functions, initialized from null initial conditions, were capable of canceling the measurement errors in less than 30 min, allowing a complete system calibration in less than one orbital period, which is a time scale relevant for the spacecraft operations and for the error drifts. Moreover, it is noted that the initial cold start trend, leading to the slower convergence of the calibration filters, was not present when the attitude determination functions were already up and running.

**Figure 17:**
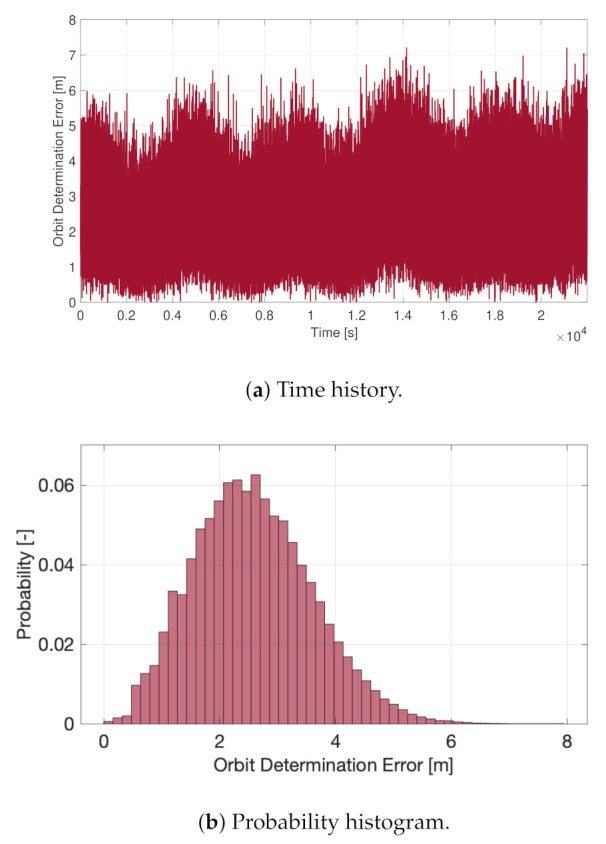
Orbit determination error.

The orbit determination functions are fundamental in satisfying the imposed mission requirements, but they also guarantee the correct on-board environmental modeling to carry out the attitude determination calculations. In fact, the wrong position estimation may impact the computation of the Sun direction and of the geomagnetic field vector in the inertial reference frame, with a direct negative consequence on the attitude determination accuracy. Figure 17 shows the absolute position error of the on-board determination functions, with performance fully in line with the imposed requirements. In fact, the three-axis orbit determination error was smaller than 10 m at all times the GNSS receiver was operational. The presence of the accelerometers and of the on-board orbit propagation functions did not significantly improve the GNSS measurements, but it made the overall orbit determination block robust with respect to GNSS failures and more reliable in the case of orbital control maneuvers. In fact, in this last case, the accelerometers provided a faster and more reliable convergence to the correct new orbital states.

## 5. Final Remarks

The proposed sensor architectures guarantees the solution of the attitude determination problem with a maximum attitude knowledge error of 1 deg at $1\sigma_{a}$ and with a maximum orbital position knowledge error of 10 m at $1\sigma_{o}$. These results were achieved with commercial off-the-shelf (COTS) sensors in the class of nano-satellites. Indeed, the proposed sensor architecture has to be compliant with many constraints in terms of the mass, volume, power, computational resources, and cost budget. Specifically, the attitude determination functions were based on the Quaternion ESTimation (QUEST) algorithm processing Sun and geomagnetic field measurements. This function was combined with gyroscope data to guarantee attitude propagation during eclipses and with on-board sensor calibration functions to improve the attitude estimation performance. The orbit determination block was founded on direct GNSS measurements loosely coupled through an extended Kalman filter (EKF), with accelerometers data and with an on-board orbit propagator. This design allows the robustness of the entire navigation block with respect to GNSS failures and outage periods. Moreover, it allows a direct integration of the proposed sensor architecture in missions with orbital control capabilities. The proposed results describe the sensor performance and features of classical nano-satellite components. In particular, hardware-in-the-loop (HIL) test results were made available and discussed with respect to the selection analyses to build up the proposed sensor architecture. The initial assumptions and justifications were motivated by the design process description, which was supported by extensive testing activities summarized in this manuscript. The same test results were used to fully characterize the complete sensor architecture, in order to set up proper model-in-the-loop (MIL) verification activities demonstrating the accuracy of the full-attitude determination process. The combined verification process at the hardware and model levels allowed achieving high-quality standards in the design of nano-satellite missions. Despite this research work being based on the activities carried out for the HERMES nano-satellites, the information contained in this manuscript can be used as a reference for spacecraft with analogous sensor typologies, mission requirements, or system constraints. All of them are quite common in the rising sector of nano-satellite and CubeSat missions.

## Figures and Tables

**Table 1:** Assumed system constraints for the proposed sensor architecture.

Constraint	Value	Units	Notes
Spacecraft mass	≤10	kg	
Spacecraft volume	≤6	U	CubeSat Standard
Power budget	≤50	W	
On-board computer frequency	≤128	MHz	
Memory footprint	≤32	MB	
ADCS cost budget	≤75	k€	

**Table 2:** Memsense MS3025M gyroscope specifications.

Specification	Value	Units	Notes
Bias Instability	1.0662	deg/h	Worst Axis Value
Angle Random Walk	0.1296	$\deg/\sqrt{h}$	Worst Axis Value
Scale Factor Error	1785	ppm	Worst Axis Value
Mass	17.8	g	
Sampling Power	0.92	W	

**Table 3:** Invensense MPU3300 gyroscope specifications.

Specification	Value	Units	Notes
Bias Instability	5.3245	deg/h	Worst Axis Value
Angle Random Walk	0.7454	$\deg/\sqrt{h}$	Worst Axis Value
Scale Factor Error	5534	ppm	Worst Axis Value
Mass	≤1	g	
Sampling Power	0.0119	W	

**Table 4:** Fine sun sensor specifications.

Specification	Value	Units	Notes
Typical Measurement Error	∼0.5	deg	Calibrated
Scale Factor Error	12,500	ppm	Calibrated
Typical Measurement Error	1.5	deg	Uncalibrated
Maximum Measurement Error	3.5	deg	Uncalibrated; Worst Axis Value
Scale Factor Error	50,000	ppm	Uncalibrated; Worst Axis Value

**Table 5:** PNI RM3100 magnetometer specifications.

Specification	Value	Units	Notes
Noise Level	17	nT	Worst Axis Value
Bias	440	nT	Worst Axis Value
Scale Factor Error	4885	ppm	Worst Axis Value
Mass	7.9	g	
Sampling Power	8.5	mW	

**Table 6:** Memsense MS3025M magnetometer specifications.

Specification	Value	Units	Notes
Noise Level	144	nT	Worst Axis Value
Bias	3491	nT	Worst Axis Value
Scale Factor Error	7765	ppm	Worst Axis Value

**Table 7:** Novatel OEM719 GNSS receiver specifications.

Specification	Value	Units	Notes
Position Accuracy	1.5	m	Datasheet Declared
Velocity Accuracy	0.03	m/s	Datasheet Declared
Time Accuracy	20	ns	Datasheet Declared
Time to First Fix	39	s	Cold Start; Datasheet Declared
Mass	33.2	g	
Sampling Power	1.32	W	

**Table 8:** Memsense MS3025M accelerometer specifications.

Specification	Value	Units	Notes
Bias Instability	2.2652	μg	Worst Axis Value
Velocity Random Walk	0.00783	$\left(m/s\right)/\sqrt{h}$	Worst Axis Value
Scale Factor Error	1157	ppm	Worst Axis Value

**Table 9:** Model-in-the-loop simulation parameters.

Specification	Value	Units	Notes
Principal Inertia Moments	[0.0667 0.0669 0.0255]	${\mathrm{kgm}}^{2}$	
Simulation Time	6	h	∼ 4 Orbital Periods
Spacecraft Orbit	Equatorial	N/A	
Orbit Altitude	550	km	
Initial Attitude State	Random	N/A	
Initial Angular Velocity	μ=0 deg/s, σ=0.1 deg/s	N/A	Gaussian Distribution
Attitude Mode	Nadir Pointing	N/A	
Attitude Pointing Error	0	deg	Ideal Attitude Tracking

## Data Availability

No new data were created or analyzed in this study. Data sharing is not applicable to this article.
